# YB-1 Oncoprotein Controls PI3K/Akt Pathway by Reducing Pten Protein Level

**DOI:** 10.3390/genes12101551

**Published:** 2021-09-29

**Authors:** Antonella Delicato, Eleonora Montuori, Tiziana Angrisano, Alessandra Pollice, Viola Calabrò

**Affiliations:** Department of Biology, University of Naples Federico II, MSA-Via Cinthia, 26-80126 Naples, Italy; antonella.delicato@unina.it (A.D.); el.montuori@studenti.unina.it (E.M.); tangrisa@unina.it (T.A.)

**Keywords:** YB-1, PTEN, cold-shock proteins, proteasome, PI3K/Akt pathway

## Abstract

YB-1 is a multifunctional protein overexpressed in many types of cancer. It is a crucial oncoprotein that regulates cancer cell progression and proliferation. Ubiquitously expressed in human cells, YB-1 protein functions are strictly dependent on its subcellular localization. In the cytoplasm, where YB-1 is primarily localized, it regulates mRNA translation and stability. However, in response to stress stimuli and activation of PI3K and RSK signaling, YB-1 moves to the nucleus acting as a prosurvival factor. YB-1 is reported to regulate many cellular signaling pathways in different types of malignancies. Furthermore, several observations also suggest that YB-1 is a sensor of oxidative stress and DNA damage. Here we show that YB-1 reduces PTEN intracellular levels thus leading to PI3K/Akt pathway activation. Remarkably, PTEN reduction mediated by YB-1 overexpression can be observed in human immortalized keratinocytes and HEK293T cells and cannot be reversed by proteasome inhibition. Real-time PCR data indicate that YB-1 silencing up-regulates the PTEN mRNA level. Collectively, these observations indicate that YB-1 negatively controls PTEN at the transcript level and its overexpression could confer survival and proliferative advantage to PTEN proficient cancer cells.

## 1. Introduction

YB-1 is a DNA- and RNA-binding protein and transcription factor with an evolutionarily ancient and conserved cold shock domain [1]. In healthy tissues, YB-1 is primarily cytoplasmic, where it plays an important role in regulating various aspects of RNA biology [2]. YB-1 is a major component of translationally inactive messenger ribonucleoprotein particles (mRNPs) and is mainly responsible for the storage of mRNAs in a silent state [3].

Since its initial discovery, the Y-box binding protein 1 (YB-1) was linked to oncogenic functions and chemotherapy resistance. YB-1 is upregulated in tumors and its nuclear localization is associated with a more aggressive phenotype indicating a poor prognosis [4,5,6,7]. In response to genotoxic stress, YB-1 translocates from the cytoplasm to the nucleus [8], where it acts as a transcriptional regulator to overcome DNA damage-dependent cell cycle arrest and promote cell survival [9].

YB-1 is a direct target of the serine/threonine kinase Akt. Akt is activated by phosphorylation at Ser473. Overstimulated Akt activity in cancer cells [10] induces YB-1 phosphorylation at Ser102 and nuclear accumulation without changing the total amount of the protein. This results in reduced DNA repair in cancer cells after irradiation [3].

The nuclear accumulation of YB-1 in response to DNA damage or transcription inhibition requires a decrease in the cytoplasmic mRNA level [11]. Indeed, like Akt, YB-1 is associated with inactive mRNPs and, activated Akt relieves translational repression of the YB-1-bound mRNAs thereby facilitating translational activation of silenced mRNA species [3,12].

**Figure 1:**
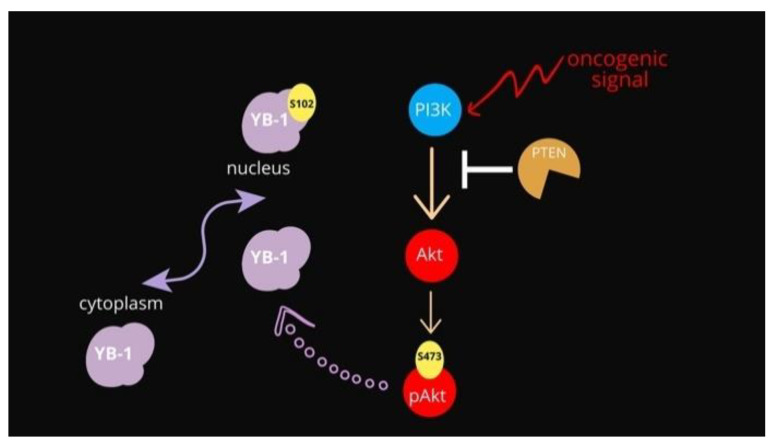
Schematic representation of the PI3K/Akt pathway overseeing YB-1 activation.

The main negative regulator of the PI3K-Akt pathway remains the PTEN phosphatase. Phosphatase and tensin homolog deleted on chromosome 10 (PTEN) is recognized as a tumor suppressor due to its negative regulation of the phosphatidylinositol-4, 5-bisphosphate 3-kinase (PI3K)/protein kinase B (Akt) signaling pathway [13], see Figure 1. The major target of PTEN is phosphatidylinositol (3, 4, 5)-triphosphate (PIP3), which is generated by PI3K and acts as a bridge to recruit 3-phosphoinositide-dependent protein kinase 1 (PDK1) and AKT to the plasma membrane, further activating AKT by phosphorylation at its T308 site [13,14]. PTEN converts PIP3 into PIP2, interrupting the interaction between PDK1 and AKT, and thus negatively mediating the activation of AKT. Apart from its membrane-bound form, nuclear PTEN has multiple functions, including the induction of cell cycle arrest by inhibiting cyclin D1 expression [15], maintenance of chromosomal stability, and DNA double-strand break repair [16].

Loss of PTEN activity has been identified in a wide spectrum of primary and metastatic neoplasms, including breast cancer [17]. This condition, which results in low or null expression of the protein, is believed to be an early oncogenic event in several tumor types [18,19]. Given the ability of Akt to physically interact and activate YB-1 oncogenic functions [3] we hypothesize that YB-1 was, in turn, able to regulate Akt by a positive control that could mirror what happens in vivo when YB-1 overexpression sustains the proliferative and survival potential of cancer cells. Very little information about YB-1 and PTEN functional crosstalk is now available. However, a significant association of YB-1 nuclear accumulation with PTEN deletion in advanced prostate tumor stages was reported by Heumann and collaborators in 2017 thus suggesting a possible reciprocal regulation between the two proteins of the PI3K/Akt pathway [20]. Here we provide compelling evidence that YB-1 can sustain Akt activation by controlling PTEN.

## 2. Materials and Methods

### 2.1. Plasmids and Reagents

The expression plasmid 3XFlag-YB-1 wt was used for the transfection and provided by Dr. Arezoo Astanehe (Abbotsford, BC, Canada). The pcDNA-GFP plasmid, used as a control, was purchased by Thermo-Fisher Scientific (Waltham, MA, USA). Sodium (meta)arsenite (NaAsO_2_, S7400, Sigma-Aldrich, St. Louis, MO, USA) and copper (II) sulfate (C1297, Sigma-Aldrich) were used to treat cell culture at 300 μM and 10 μM final concentrations, respectively. MG-132 (M8699, Sigma-Aldrich) was used as a proteasome inhibitor at 10 μM final concentration, for 4h.

### 2.2. Cell Cultures

Human embryonic kidney cells (HEK293T) and HaCaT (human spontaneously immortalized keratinocytes from adult skin) were purchased from Cell Line Service (CLS, Germany) and cultured in a humidified incubator at 37 °C and 5% CO2 in DMEM High glucose (Gibco BRL, Grand Island, NY, USA) supplemented with 10% Fetal Bovine Serum (Gibco BRL), 1% L-glutamine (Gibco BRL) and 1% Pen-Strep solution (Gibco BRL). Cells were routinely checked for mycoplasma contamination, using a mycoplasma detection kit (Abcam, Quebec, QC, Canada).

To increase HEK293T adhesion to glass/plastic surfaces, plates were treated with poly-D-lysine (0.1 mg/mL, P7405, Sigma-Aldrich) before seeding cells.

### 2.3. Immunoblotting Analysis

For total protein extraction 2.5 × 10^5^ cells were seeded in 6-well. After 48 h, cells were harvested in lysis buffer (50 mM Tris-HCl pH 7.5, 5 mM EDTA, 150 mM NaCl, 1% NP-40, 0.5% sodium deoxycholate) with the addition of 1 mM phenylmethylsulfonyl fluoride and protease and phosphatase inhibitor cocktail (Sigma-Aldrich). Cells were detached with a scraper and left on ice for 30′. Then extracts were clarified by centrifugation at 13,200 rpm for 30′ at 4 °C. The amount of protein in the samples was determined by the Bio-Rad protein assay (Bio-Rad, Milan, Italy).

After the addition of Laemmli buffer (Sigma-Aldrich) samples were boiled at 100 °C for 5 min and resolved by SDS- polyacrylamide gel electrophoresis (SDS-PAGE). About 20 μg of total extracts were separated by SDS-PAGE.

Proteins were then transferred to a polyvinylidene difluoride membrane (PVDF, Millipore) using a Mini trans-blot apparatus (Bio-Rad) according to the manufacturer’s instructions. The PVDF membrane was blocked in 5% *w/v* milk buffer (5% *w/v* non-fat dried milk, 50 mM Tris, 200 mM NaCl, 0.2% Tween 20) and incubated overnight at 4 °C with primary antibodies diluted in 5% *w/v* milk or bovine serum albumin (BSA) buffer according to the manufacturer’s instructions. Following three washes with TBST (Tris-buffered saline, 0.1% Tween), the blots were incubated for 1 hour at RT with HRP-conjugated secondary antibodies (Sigma-Aldrich). Proteins were visualized by enhanced chemiluminescence (ECL, Bio-Rad) and analyzed by Quantity One W software of ChemiDoc TM XRS system (Bio-Rad).

Band intensities were quantified by ImageJ Software (http://imageJ.nih.gov/ij/, accessed on 21 August 2021, free software, downloaded from the NIH, Bethesda, MD, USA), normalized respect loading control and reported as fold enrichment to the control sample.

### 2.4. Antibodies

The primary antibodies used are: anti-YB-1 raised against the region 1 to 100 of YB-1 protein (12148 Abcam, Cambridge, UK); anti-Actin (8432 Santa Cruz, Dallas, TX, USA); anti-PTEN (Cell Signaling, Danvers, MA, USA, 9559S); anti-Phospho-Akt (Ser473) (193H12) (Cell Signaling, 4058S); anti-Akt (Cell Signaling, 2920S).

### 2.5. Transfections and RNA Interference

Cells were transfected using Lipofectamine 2000 (Life Technologies, Carlsbad, CA, USA) according to the manufacturer’s recommendations. Briefly, cells were seeded at 70–80% confluence (2.5 × 10^5^) in 6-well and transiently transfected with plasmids at a concentration of 300 ng for 48h. MG-132 was added to the cells at the concentration of 10 μM 4 h before the end of transfection.

YB-1 transient silencing was carried out with IBONI YB-1 small interfering (siRNA) pool (RIBOXX GmbH, Radebeul, Germany) as a pool of 3 different siRNAs and RNAiMAX reagent (Life Technologies), according to the manufacturer’s recommendations. Cells were seeded at 70–80% confluence (2.5 × 10^5^) in 6-well and transiently silenced with IBONI YB1-siRNA at 150 nM final concentration.

Negative Control siRNA, provided by RIBOXX (Germany) was used as a negative control.

YB-1 guide and passenger sequences:

*h* YBX-1 guide (5′-3′):

UUUAUCUUCUUCAUUGCCGCCCCC

UUAUUCUUCUUUAUGGCAGCCCCC

UAUUUGAUGACCACACCAGCCCCC

*h* YBX-1 passenger (5′-3′):

GGGGGCGGCAAUGAAGAAGAUAAA

GGGGGCUGCCAUAAAGAAGAAUAA

GGGGGCUGGUGUGGUCAUCAAAUA

### 2.6. Co-Immunoprecipitation

For co-immunoprecipitations (Co-IP) 2 × 10^6^ HEK293T cells were seeded in poly-D-lysine pre-treated 100-mm dishes; the day after Dynabeads Protein A (Invitrogen) were incubated with antibodies against YB-1, 3 μg for 1.5 mg of protein extract for 10′ at room temperature in rotation after protein extract was incubated with the Dynabeads-Ab complex overnight at 4 °C. Immunoglobulin G (IgG) 3 μg for 1.5 mg of protein extract was used as a negative control. Immunocomplexes were resolved with SDS-PAGE; immunoblot was performed with anti-PTEN antibody and anti-YB1 antibody.

### 2.7. Quantitative Real Time-PCR

Total RNA was extracted with Trizol reagent (Gibco) according to the manufacturer’s instructions. Reverse transcription was performed using All-In-One 5X RT MasterMix (Abcam). AriaMx Real-Time PCR System (Agilent Technologies, Santa Clara, CA, USA) and Brilliant HRM Ultrafast Starter Pack were used. Quantitative relative expression was calculated according to the 2^−ΔΔCT^ method (Delta CT method) normalizing to *rpl0* mRNA expression. Sequences of primers used:

PTEN *For:* CTCAGCCGTTACCTGTGTGT

PTEN *Rev*: AGGTTTCCTCTGGTCCTGGT

YB-1 *For*: CGACCAGACTCTCATCCTGC

YB-1 *Rev:* TTTGATGACCACACCAGGCA

RPL0 *For*: GACGGATTACACCTTCCCACTT

RPL0 *Rev:* GGCAGATGGATCAGCCAAGA

### 2.8. Statistical Analysis

Statistical analyses were performed using GraphPad Prism (version 8.1.2, GraphPad Software Inc., San Diego, CA, USA). Data were presented as the mean ± standard deviation and analyzed for statistical significance using one-way or two-way analysis of variance (ANOVA) and multiple comparisons. For all tests, *p* < 0.05 was considered to indicate a statistically significant difference. To report *p*-values, the *New England Journal of Medicine* (NEJM) decimal format was used; differences were considered statistically significant at * *p* < 0.033, ** *p* < 0.002 and *** *p* < 0.001.

## 3. Results

Firstly, we wondered whether there was a direct functional relationship between YB-1 and PTEN. We hypothesized that YB-1 could control PTEN protein level. To address this point, we decided to analyze the effect of YB-1 downregulation on PTEN protein intracellular level.

**Figure 2:**
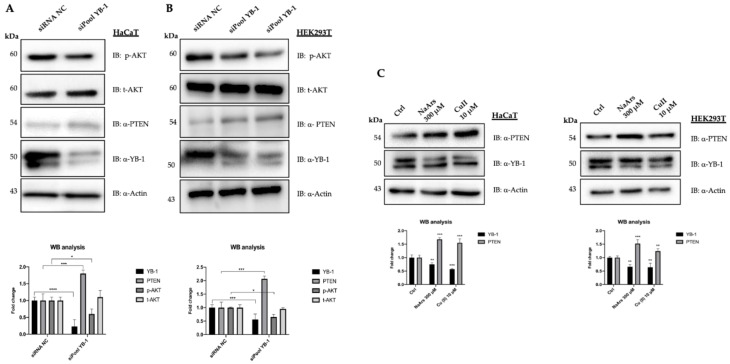
YB-1 controls PTEN levels in HaCaT and HEK293T cells. (**A**) HaCaT cells were transfected with 10 nM final siRNA-poolYB-1 or siRNA-Negative Control (NC). The effect on PTEN intracellular levels was evaluated 48 h post-transfection by western blot on whole protein lysates probed with anti-PTEN antibodies. Immunoblots were also probed with antibodies against YB-1, pAkt^S743^, tAKT and actin as a loading control. (**B**) HEK293T cells were transfected with 10 nM final siRNA-NC or siRNA-poolYB-1 (duplicated). Whole protein lysates were collected and analysed 48 h after transfection as described in (**A**); (**C**) HaCaT and HEK293T cells were treated with NaArs and Cu (II) for 2 h to induce oxidative stress. Cell lysates were analyzed by immunoblot with antibodies against PTEN, YB1 and actin used as a loading control. For comparison, siRNA-transfected extracts were used. Statistical analyses were performed using 2-way ANOVA and Sidak’s multiple comparison or Dunnett’s multiple comparisons test. Levels of significance between points of expression are indicated (*** *p* < 0.001, ** *p* < 0.01, * *p* < 0.05).

To this aim, we used PTEN proficient immortalized HaCaT and HEK293T cells, showing robust expression of endogenous YB-1 resembling actively proliferating pre-malignant cells. YB-1 expression was depleted by RNAi and PTEN levels were checked by immunoblot. Control (siRNA NC) and YB-1 depleted cells (siPool YB-1) were collected 48h after transfection and analysed by immunoblot. As shown in Figure 2A we observed a clear increase of PTEN levels in YB-1 depleted HaCaT cells compared to the control with a concomitant reduction of pAkt^S743^ (Figure 2B) in line with a potential role of YB-1 in enhancing Akt phosphorylation at S743 by directly impinging on PTEN/PI3K pathway. An even more evident increase of PTEN protein levels was observed in HEK293T cells upon YB-1 depletion (Figure 2B). Interestingly, an increase of PTEN protein level was also observed in total extracts from HaCaT and HEK293T cells whose level of YB-1 was reduced by oxidative stress stimuli such as that with NaArs and Cu++ treatments (Figure 2C), as we have previously shown [21].

**Figure 3:**
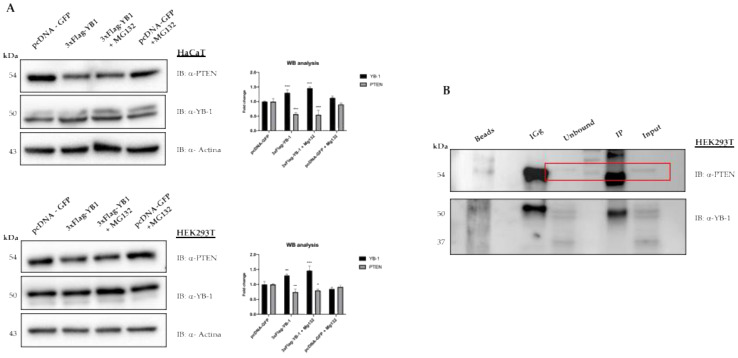
YB-1 mediated reduction of PTEN levels requires neither the proteasomal activity nor the physical interaction between the two proteins. (**A**) HaCaT cells (top panel) and HEK293T cells (lower panel) were transfected with 3XFlag-YB-1 plasmid or pcDNA-GFP as a control. Effect on PTEN intracellular levels was evaluated 48 h post-transfection by western blot on whole protein lysates probed with anti-YB-1, anti-PTEN, and anti-actin as a loading control. 10 μM MG132 for 4 h before the end of transfection was used to inhibit proteasome activity. (**B**) HEK293T protein extracts were immunoprecipitated with anti-YB-1 antibodies. Immunocomplexes were subjected to western blot and revealed using antibodies against PTEN. PTEN signal in unbound and input are highlighted in the rectangle box. Statistical analysis was performed using 2-way ANOVA and Sidak’s multiple comparison test. Levels of significance between points of expression are indicated (*** *p* < 0.001, ** *p* < 0.01, * *p* < 0.05).

We then decided to look next at the effect of YB-1 overexpression on PTEN protein levels. To this aim, we transfected YB1 expression vector in HEK293T or HaCat cells treated or not with the proteasome inhibitor MG132. At 48 h after transfection, cells were collected, and the extracts were analysed by immunoblot. As shown in Figure 3A we observed a moderate but significant reduction of PTEN protein level, compared to the control, that was not rescued by MG132 treatment thus suggesting that it does not occur through a proteasome-dependent mechanism. We also tested for a possible interaction between YB-1 and PTEN proteins by co-immunoprecipitation. However, as shown in Figure 3B, YB-1 antibodies were unable to immunoprecipitate endogenous PTEN.

**Figure 4:**
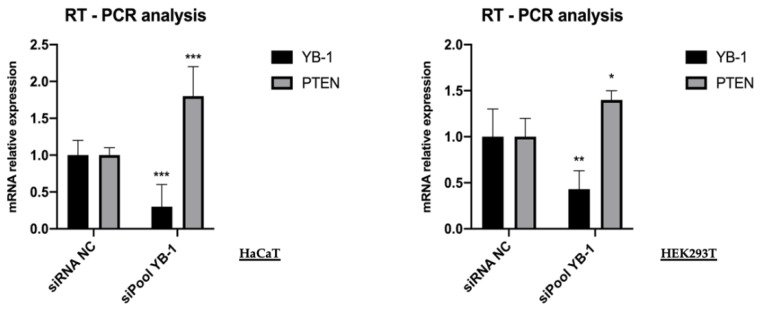
YB-1 depletion increases the level of PTEN transcript. HaCaT and HEK293T cells were transfected with 10 nM final siRNA-poolYB-1 or siNC; the effect on PTEN mRNA level was evaluated 48h post-transfection by qRT-PCR analysis. Relative mRNA levels were plotted on the y-axis and siRNA employed are indicated on the x-axis. RPL0 ribosomal protein mRNA was used for normalization. Statistical analyses were performed using 2-way ANOVA and Sidak’s multiple comparison test. Levels of significance between points of expression are indicated (*** *p* < 0.001, ** *p* < 0.01, * *p* < 0.05).

Given the importance of YB-1 function in the control of RNA metabolism, we wished to explore whether YB-1 depletion was able to alter the level of PTEN mRNA. Therefore, we depleted HaCaT and HEK293T cells of YB-1 using the siRNA pool against YB-1 (siPoolYB-1) [21]. Total RNA was extracted and subjected to RT-qPCR. As shown in Figure 4, compared to the control sample the mRNA level of YB-1 was reduced to 0.25 in HaCaT and 0.40 in HEK293T cells while PTEN mRNA was increased to 1.75 in HaCaT and 1.5 in HEK293T cells. 

This result showed that the effect of YB-1 on PTEN was primarily at mRNA level. It is important to remind that PTEN functions in a dosage-dependent manner during tumor development and that moderate PTEN reduction, without complete loss, has been reported to activate the PI3K/Akt pathway and to be associated with chemoresistance and cancer progression [22,23].

## 4. Conclusions

**Figure 5:**
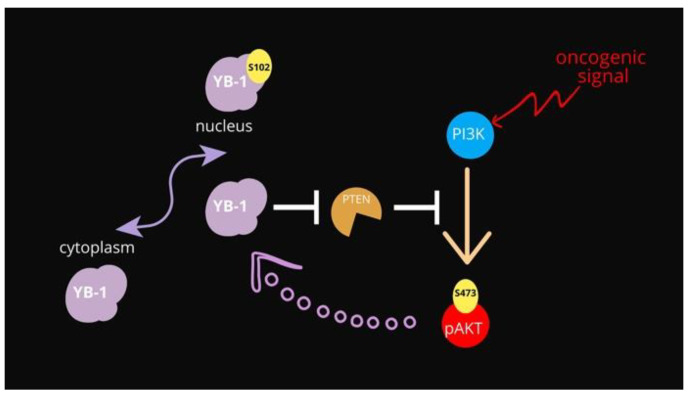
Schematic representation of the proposed functional relationships among YB-1, PTEN and Akt. In normal conditions, YB-1 is mainly cytoplasmic. The Akt-dependent phosphorylation of YB-1 at S102 promotes its translocation to the nuclear compartment promoting cell survival.

The data presented here show evidence that YB-1 controls PTEN protein levels by acting at the transcript level. Our data are in line with what is already known regarding the function of both proteins, although a direct functional link between PTEN and YB-1 has never been assessed. At the functional level increased expression of YB-1 can restrain PTEN expression thus enforcing Akt activation in premalignant cells (Figure 5). Akt, in turn, increases the pro-proliferative and pro-survival activities of YB-1 by inducing YB1 phosphorylation at S102 thereby promoting its nuclear translocation.

The PTEN level appears to be tightly controlled both transcriptionally and post-transcriptionally [24]. Some oncogenic microRNA and ncRNAs have been found to target PTEN mRNA and regulate malignant progression [25], ncRNAs including lncRNAs and miRNAs act alone or interact with each other to regulate PTEN expression and it has recently been proposed that some of the oncogenic effects of YB-1 in breast cancer may be mediated through its interactions with sncRNAs [26].

Although the precise mechanism through which YB-1 controls PTEN mRNA level remains to be determined, our data suggest the existence of a positive feedback loop between YB-1 and Akt, reinforcing each other, probably occurring at an early step in cancer progression and conferring a selective advantage to premalignant cells. Elucidation of the details about how YB-1 downregulates PTEN expression may provide novel insights into the regulation network of PTEN, which could suggest possible anticancer strategies focusing on targeting both YB-1 and the PI3K/Akt pathway.
